# Supplementary material for: Bio-Efficacy of Chrysoeriol7, a Natural Chemical and Repellent, against Brown Planthopper in Rice
Source: Int J Mol Sci. 2022 Jan 28;23(3):1540. doi: 10.3390/ijms23031540 (PMC8836193; doi:10.3390/ijms23031540)
Supplement: Supplementary file 1 [file ijms-23-01540-s001.zip › ijms-1533026-supplementary.pdf]

Supplementary Materials

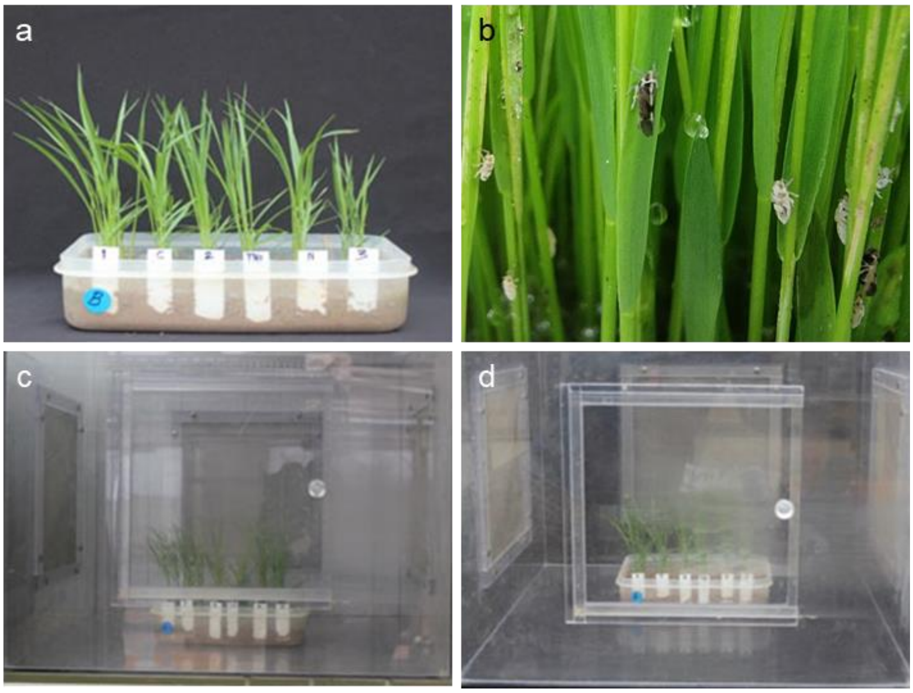

Figure S1. BPH rearing for the BPH infection to rice. (**a**) Plant materials. BPH-resistant population: Samgang, SNDH29, and SNDH30. BPH-susceptible population: Nagdong, TN1, and SNDH11. (**b**) BPH is reared in the cage for the BPH infection in each population. (**c**) Control group. (**d**) Experiment group. After BPH infection at the seedling stage was sampled for 1 day, 2 days, and 3 days.
